# Effect of neonatal breast crawl on breastfeeding: a prospective cohort study

**DOI:** 10.3389/fped.2023.1186585

**Published:** 2023-06-08

**Authors:** Yan Pang, Xin Wang, Huimin Li, Suhua Tu

**Affiliations:** ^1^ Department of Nursing, Chengdu Women and Children's Central Hospital, Chengdu, China; ^2^ Department of Obstetrics, The Affiliated Hospital of Southwest Medical University, Luzhou, China

**Keywords:** breast crawl, breastfeeding, autonomous milk seeking, lactation, midwifery

## Abstract

**Aim:**

To analyse the effect of breast crawl on neonatal breastfeeding within 5 months of delivery.

**Design:**

Prospective Cohort Study.

**Methods:**

Neonates were divided into successful and failed groups, according to whether the newborn crawled to the breast and began sucking for the first time within 1 h after delivery. The initiation of lactation and breastfeeding duration of the two groups were analysed at 24 h, 48 h, 72 h, the feeding practices were followed-up on the 7th day, 42nd day, and 5th month in order to explore the long-term benefits of breast crawl on breastfeeding.

**Results:**

A total of 163 neonates were included. The initiation time and the duration of first feeding, the lactation initiation in the successful group was earlier, the scores of first and in-hospital breastfeeding scales were higher.

**Public Contribution:**

Breast crawl is the preferred method for mothers to begin breastfeeding. The delivery room is the place where the first breast crawl occurs immediately after delivery. The midwife is the key person to protect this valuable behavior. Therefore, the midwife needs provide valuable opportunities for the breast crawl of the newborn and promote this behavior.

## 1. Introduction

Epigenetic genomic imprinting occurs actively during the period from birth to two years of age (1), which can affect the subsequent nutrition and health of infants, and has become the basis for promoting the overall development of the population, improving the quality of human capital, and ensuring sustainable development of the society (2). As an essential unadulterated food, breast milk is a personalised medicine for infants, which can prevent the occurrence of certain infectious and specific diseases, such as cardiovascular diseases, leukaemia, necrotising enterocolitis, celiac disease, and inflammatory bowel disease (3), thereby reducing the hospital revisit rate of infants. Evidence from *The Lancet* has also indicated that non-breastfeeding are associated with low intelligence in infants and young children and national economic losses, which was estimated to be $302 billion per year, accounting for 0.49% of the world's gross national income (4). Therefore, breastfeeding is one of the most important measures to ensure the healthy growth of infants and young children. Despite its established benefits, breastfeeding is no longer the norm in many places. In the past 20∼30 years, breastfeeding has been replaced by formula milk. With the improvement of maternal and childcare policies, the concept of natural parenting has gradually gained importance. However, lacking of evidence-based concept and skills of breastfeeding have made it difficult for many mothers to breastfeed. According to the Breastfeeding Promotion Action Plan (2021–2025), the exclusive breastfeeding rate of infants within 6 months of birth should reach at least 50%. However, the survey data in 2019 showed that the exclusive breastfeeding rate during this period was only 29.2%, which is still far below the target (2). Breastfeeding is a process of coordination between the mother and the baby. The mother's lactation function are not only related to her own anatomy, physiology, health, but also affected by the environment and baby's birth situation. In other words, breastfeeding is not something that mothers can do on their own.

Breast crawl makes breastfeeding more natural by tapping into the neonate's instinct to find milk autonomously. Breast crawl refers to the natural crawling toward the mother's breast; in this process of skin-to-skin contact between mother and infant without external interference, the neonate locates the nipple and self-attaches for the first feeding until the first breastfeeding (5). Breast crawl sequences include nine stages: birth cry, relaxation, awakening, activity, resting, crawling, familiarisation, sucking, sleeping (6), are essential for newborn survival behaviour (7), conducive for stable vital signs and behavioural states, forming multi-sensory stimulation and early attachment signals, promoting the maturity of parasympathetic nerves, and increasing newborn milk intake capacity. The loss of breast crawl may cause the neonate to lose some innate abilities, thereby affecting the structural or functional integrity of the brain and body (8). In 1998, Klaus introduced the concept of “breast crawl,” and the United Nations International Children's Emergency Fund (UNICEF) recommended that “breast crawl is the preferred method for mothers to begin breastfeeding their neonates” (5). The American Academy of Paediatrics (AAP) recommended that “all healthy infants should begin skin contact with their mothers immediately after delivery until the first feeding occurs” (9). Suffice to prove that breast crawl promotes breastfeeding. However, in clinical work, it was found that medical staff did not pay attention to the process of newborn breast crawl, but still used routine mother-infant skin contact as the main method, that is, let newborn passively hold and suck the nipple after the occurrence of mammary matting behavior (10), while ignoring the instinctive mammary matting behavior and ability of newborn, and passive nipple containing neonates are prone to breast and nipple rejection, which leads to breastfeeding failure (11). Meanwhile, domestic research on this topic is still in initial stage. Till date, only the time and proportion of newborn breast crawl (12) and the effects of short-term breastfeeding have been studied (13).

Breastfeeding is a persistent event, thus, this study used breastfeeding as an observational indicator to explore the long-term effects of crawling on breastfeeding.

## 2. Methods

### 2.1. Study design

The present study was a prospective cohort study in China. Convenience sampling was used to select women and their neonates who underwent vaginal delivery in a Grade three A hospital from Nov 2019 to Sept 2020 according to the admission criteria. Inclusion criteria included: (1) low-risk pregnant women, (2) maternal breastfeeding intention, (3) puerpera who provided informed consent and were willing to participate and cooperate with the study, (4) women with vaginal delivery of full term children, and an Apgar score of ≥8 in neonates. Exclusion criteria were: (1) puerpera who refused breast crawl and neonates who needed medical intervention. Criteria for suspension and loss of follow-up included: (1) abnormal vital signs of mother and infant during crawling, (2) voluntary withdrawal from the study, and loss of contact during follow-up. The participants were informed of their right to decline or withdraw at any time during the study.

### 2.2. Sample sizes

Sample size was determined according to the follow-up cohort study:

$$n=\frac{{\left[Z_{\alpha }\times \sqrt{2\bar{\;p}}\bar{q}+Z_{\beta }\sqrt{\;p_{0}q_{0}+p_{1}q_{1}}\right]}^{2}}{{\left(p_{1}-p_{0}\right)}^{2}}$$

A literature review was conducted and similar mother–infant skin contact studies were selected, where it was found that the rate of exclusive breastfeeding in the follow-up of the intervention group was 81.67%, whereas that of the control group was 65.00% (10). The sample size was calculated to be 140 cases, and 156 cases were needed after considering a 10% loss of follow-up.

### 2.3. Study tool

The Breastfeeding Assessment Tool (BAT) (14) developed by Matthews for measuring breastfeeding was used. This tool includes four dimensions: ready to feed (the baby's reaction when the feeder picks up the baby), rooting reflex (the specific performance of the baby when the cheek touches the nipple), latching-on (the sucking situation of the baby during feeding), and sucking (the mouth of the baby is wide and contains the nipple and areola, lower lip eversion, the tongue is ladled around the breast). Each dimension contains four items, in which the score of each item is 0∼3, whereas the total score is 0∼12. A total score of 8 or more indicates successful breastfeeding, a score below 10 indicates suboptimal breastfeeding behaviour, and a score below 8 indicates failed breastfeeding. The consistency coefficient of the scale in domestic studies was 0.97 (15), indicating good validity. The Cronbach's *α* coefficient in this study was 0.827.

### 2.4. Setting

The expectant mothers were informed of the purpose and methods of the study before conducting the study. Under the supervision of the head nurse of the department, the researchers trained relevant personnel who were designated their respective responsibilities. The specific processes were as follows: (1) Adjusted the room temperature in the delivery room to 26∼28°C. Immediately after birth, the exposed newborn lied prone on the abdomen of the mother. Dried the newborn within 20∼30 s (without wiping the hands and breast of the puerpera). After the umbilical cord stopped pulsating for 1∼3 min, the cord was broken. (2) After monitoring the newborn without abnormal conditions, put the newborn in a prone position in front of the mother's chest,eyes level up to the nipple position, face to one side, so that the newborn's toes touched the mother's uterus or pubic symphysis. (3) Covered the back of the newborn with baby clothes and towels and worn a hat. Guided the mother to hold the soles of the feet with one hand and place the other hand on the newborn's back to ensure that the newborn does not slip. (4) In the process of skin contact, when the newborn showed breast searching behaviours, such as crawling, mouth and tongue movements after finding milk, there should be no interventions to disrupt its crawling, until the newborn independently positioned herself/himself onto the breast and started breastfeeding for the first time. Medical intervention should be carried out immediately if the mother and child develop abnormal conditions. (5) During the crawling process, researchers actively communicated with mothers and conducted breastfeeding health education. The neonates were divided into successful and failed groups according to whether the newborn crawled to the breast and began sucking within 1 h. Neonates in the failure group who still showed foraging signs (drooling, opening mouth, eating hand, stretching arm) after 1 h, continuing to observe until they first began autonomous feeding. If the newborn entered the sleep phase directly, the midwife or researcher would help the newborn hold the nipple and complete the first breastfeeding. Follow-up was performed within 72 h postpartum and 5 months after discharge.

### 2.5. Evaluations

(1) Breastfeeding capacity: BAT was used to measure the overall breastfeeding score and feeding success of the neonates. It was measured at first feeding and during hospitalisation.
(2) Initiation time of lactation (16): Defined as maternal breast distention time after the end of childbirth. If breast fullness occurred within 72 h, it was defined as lactation initiation, Vice versa, it was defined as delayed onset of lactogenesis. The maternal were followed-up at 24 h, 48 h, and 72 h postpartum.
(3) Feeding practices: Included exclusive, mixed, and artificial feeding. Exclusive breastfeeding (EBF) meant the infant only fed on breast milk and not fed any other food or beverage or even water except drugs, vitamins, or minerals under medical indications. Mixed feeding was defined as feeding formula or a breastmilk substitute in addition to breast milk. Artificial feeding meant no breastmilk was fed; only formula or a breastmilk substitute was provided. The “24 h retrospective method” was used to investigate the feeding situation of the infants from the time point of investigation to the past 24 h (17). Since feeding practices were more likely to change within 7 days after delivery (18), as 42 days after delivery is considered as puerperal, and as maternity leave is usually for 5 months, follow-up of the feeding practices were followed-up at 24 h, 48 h, 72 h, 7 days, 42 days, and 5 months postpartum.

### 2.6. Statistics

The SPSS 24.0 was used for statistical analysis of the data. When the measurement data were in line with normal distribution, mean ± SD was used for description, and *t* test was used for comparison, whereas the median and quartile Median (IQR) were used for description, and the Mann–Whitney U rank-sum test was used for comparison. Counting data were described using frequency and percentage, and compared using the Chi-square test or Fisher's exact probability method, whereas stratified analysis was used to compare the conditions related to lactation initiation of women in successful and failed groups under different parity so as to control confounding factors. Due to the low rate and random loss to follow-up in this study, the data lost to follow-up were directly excluded. The level for statistical significance was set at *p* ≤ .05 (two-tailed).

### 2.7. Quality control

In order to emphasis the autonomy of the neonates during crawling, the relevant personnel did not interfere with the crawling process, such as the changing positions, holding the infant for more than 10 min, and inserting the nipple into the infant's mouth within 1 h. In order to continue the study and follow-up and help the postnatal mothers deal with nursing problems, in addition to the telephonic follow-up, a WeChat group was also established to guide the nursing women for timely addressal of medical problems. In the data entry stage, Epidata 3.0 was used for data entry by two people, and a consistency test was conducted. After data entry, 10% of the data were randomly selected for verification, and a statistical analysis was conducted to ensure that the data were correct.

### 2.8. Ethics

This study adhered to the ethical principles of benefit, non-harm, informed consent, and confidentiality. All participants were assured that the observations made were safely. Data collection was approved by the ethics committee of the hospital and informed consent was provided by the participants. During data collection, participants could voluntarily withdraw from the study. Participants were assured that the data collected would be kept strictly confidential.

## 3. Results

A total of 163 effective samples were finally obtained: 94 successful (57.7%) and 69 (42.3%) failed breast crawl cases. On the 7th day, 42nd day, 5th month, 1, 5, and 7 cases were lost to follow-up, respectively.

### 3.1. Participant characteristics

Maternal age was 28.94 ± 3.92 years, body mass index was 26.00 ± 3.55 kg/m^2^, 87 women were primiparous, and 76 women were multiparous. The gestational age of the neonate was 275.42 ± 7.39 days, body weight was 3,244.75 ± 355.98 g, and body length was 50.24 ± 4.13 cm, in which 85 boys and 78 girls were included. There was a statistical difference in parity between the two groups, and the failure rate of neonatal crawling in primiparous women was higher than that in multipara (*χ*^2 ^= 5.194, *P = *0.023).

### 3.2. Neonatal breastfeeding time between the two groups

There was no statistically significant difference in the time of initiation of breast-seeking behaviour between the two groups (*P = *0.154). Compared with the failure group, the initiation time of first feeding in the successful group was earlier, the duration of first breastfeeding was longer (*P *< 0.001) (Table 1).

**Table 1:** The start time of first breastfeeding, and the duration of first breastfeeding between the two groups [median (IQR), Min].

Time	Successful (*n *= 94)	Failed (*n *= 69)	Z	P
Initiation of breast-seeking behaviour	15 (9–23)	16 (11–28)	−1.427	0.154
Start of first feeding	51 (45–57)	65 (45–73)	−4.527	<0.001
Duration of first lactation	6 (53–70)	44 (30–55)	−6.214	<0.001

### 3.3. The ability to obtain breast milk between the two groups

#### 3.3.1. Overall BAT scores between the two groups

The BAT scores in the successful group were higher than failed group during the first feeding and hospitalisation(*P* < 0.001) (Table 2). The overall success rate of first feeding was 89.4% in the successful group, whereas it was 50.7% in the failed group. The successful group had fewer neonates with suboptimal breastfeeding for the first time and during hospitalisation than the failed group(*P *< 0.01) (Table 3).

**Table 2:** Overall BAT scores between the two groups[median (IQR)].

BAT scores	Successful (*n *= 94)	Failed (*n *= 69)	Z	P
First feeding	9 (10–11)	6 (8–9)	−6.593	<0.001
Feeding during hospitalisation	8 (10–11)	7 (9–10)	−4.069	<0.001

**Table 3:** Successful breastfeeding between the two groups [*n* (%)].

Measured time	BAT scores	Successful (*n *= 94)	Failed (*n *= 69)	$\chi^{2}$	P
First feeding	<8	10 (10.6)	34 (49.3)	38.618	<0.001
	8∼9	31 (36.2)	24 (34.8)		
	≥10	53 (53.2)	11 (15.9)		
Feeding during hospitalisation	<8	16 (17.0)	24 (34.8)	13.685	0.001
	8∼9	21 (22.3)	23 (33.3)		
	≥10	57 (60.7)	22 (31.9)		

### 3.4. Lactation initiation between the two groups

#### 3.4.1. Overall lactation initiation between the two groups

The number of lactation initiations in the successful group was greater than the failed (*P *= 0.006), and the successful group had more lactation initiations within 72 h postpartum than the failed (*P *= 0.040) (Tables 4, 5).

**Table 4:** Overall lactation initiation between the two groups [*n* (%)].

	Successful (*n *= 94)	Failed (*n *= 69)	$\chi^{2}$	P
Lactation initiation	74 (78.7)	40 (58.0)	8.151	0.006
Delayed onset of lactation	20 (21.3)	29 (42.0)		

**Table 5:** Lactation initiation days between the two groups [*n* (%)].

	Successful (*n *= 94)	Failed (*n *= 69)	$\chi^{2}$	P
Within 24 h	7 (7.4)	3 (4.3)	8.324^a^	0.040
24∼48h	27 (28.7)	14 (20.3)		
49∼2h	40 (42.6)	23 (33.3)		
After 72 h	20 (21.3)	29 (42.0)		

^a^ Fisher's exact probability test.

#### 3.4.2. Lactation initiation between the two groups using stratified analysis based on parity

In primiparas, the success of breast crawl had no effect on lactation initiation (*P *= 0.143), but in multipara, the number of lactation initiations in the successful group was significantly higher than the failed group (*P *= 0.025) (Table 6); however, there was no statistical difference in the effect of breast crawl on lactation initiation time among different parities (*P > *0.05) (Table 7).

**Table 6:** Lactation initiation between the two groups under different parity.

Parity (number)	Groups (total number)	Number of lactation	$\chi^{2}$	P
Primary (87)	Successful (43)	30	2.140	0.143
	Failed (44)	25		
Multiparity (76)	Successful (51)	44	5.008	0.025
	Failed (25)	16		

**Table 7:** Lactation initiation days between the two groups at different parities.

Parity (number)	Time period (total number of started by the two groups)	Number of lactation in successful	$\chi^{2}$	P
Primary (87)	Within 24 h (4)	2	2.347^a^	0.511
	25∼48 h (12)	7		
	49∼72 h (38)	21		
	After 72 h (33)	13		
Multiparity (76)	Within 24 h (6)	5	5.133	0.151
	25∼48 h (29)	20		
	49∼72 h (25)	19		
	After 72 h (16)	7		

^a^ Fisher's exact probability test.

### 3.5. Feeding practices between the two groups within 72 h after delivery

There were 71 (43.6%), 62 (38.0%) and 73 (44.8%) neonates in the two groups who were exclusively breastfed within 3 days after delivery, respectively. There were statistically significant differences in feeding practices within 48 h after delivery, and the number of neonates in the successful group who were breast-fed was higher than those who were artificially fed (*P < *0.01) (Table 8).

**Table 8:** Feeding practices between the two groups within 72 h after delivery [*n* (%)].

	Feeding practice	Successful (*n *= 94)	Failed (*n *= 69)	$\chi^{2}$	P
Within 24 h	Exclusive	47 (50.0)	24 (34.8)	9.362	0.009
	Mixed	36 (38.3)	24 (34.8)		
	Artificial^b^	11 (11.7)	21 (30.4)		
25∼48 h	Exclusive	42 (44.7)	20 (29.0)	9.622^a^	0.008
	Mixed	48 (51.1)	37 (53.6)		
	Artificial	4 (4.3)	12 (17.4)		
47∼72 h	Exclusive	42 (44.7)	31 (44.9)	1.914	0.384
	Mixed	41 (43.6)	25 (36.2)		
	Artificial	11 (1.7)	13 (18.9)		

^a^ Fisher's exact probability test.

^b^ Artificial feeding within 24 h was defined as: Except for the first breastfeeding during breast crawl, formula feeding was used for the rest of the period.

### 3.6. Neonatal breastfeeding practices between the two groups during follow-up after discharge

The overall exclusive breastfeeding rates at 7 days, 42 days, and 5 months postpartum were 61.7%, 64.6%, and 55.8%, respectively. There was no statistical difference in the feeding patterns between the two groups during the follow-up period (*P > *0.05) (Table 9).

**Table 9:** wFeeding practices between the two groups during follow-up [*n* (%)].

Postpartum	Feeding routine	Successful	Failed	Total rate of EBF	$\chi^{2}$	P
7 days	Exclusive	59 (63.4)	41 (59.4)	61.7	0.289	0.865
	Mixed	26 (28.0)	21 (30.4)			
	Artificial	8 (8.6)	7 (10.2)			
42 days	Exclusive	60 (67.4)	42 (60.9)	64.6	1.656^a^	0.470
	Mixed	27 (30.3)	23 (33.3)			
	Artificial	2 (2.3)	4 (5.8)			
5months	Exclusive	49 (53.8)	38 (58.5)	55.8	1.596	0.450
	Mixed	29 (31.9)	15 (23.0)			
	Artificial	13 (14.3)	12 (18.5)			

^a^ Fisher's exact probability test.

## 4. Discussion

To help neonates adapt to life smoothly and improve the status of breastfeeding, China has introduced a series of early health care technologies for neonates. The existing relevant studies mostly focus on the skin contact between mother and baby, and breast crawl is seen only as a foraging symptom in these studies. However, breast crawl in the real sense emphasises the autonomous breast-seeking behaviour of neonates; thus, studies focusing on this aspect, such as the influence of successful crawl on the short- and long-term outcomes of breastfeeding, deserve further exploration. Therefore, this study focused on the above issues and concluded that 57.7% of neonates could start sucking within 1 h after delivery under autonomous milk seeking, and crawl successful only had a positive significance for breastfeeding in the near term.

### 4.1. Effect of successful breast crawl on time associated with breastfeeding

The initiation time of first feeding in the successful group was earlier than that in the failed group, which was related to neurobehavioural inhibition in postnatal neonates by many factors during labour such as the total length of labor, the time between rupture of membranes and delivery,use fentanyl or oxytocin during labor (12, 19, 20). In this study, the initiation time of the first feeding was much later than 25 min, as described by Li (21). This was because our study emphasised the newborn's independent milk-seeking ability, which was different from the method of “instructing the mother to start breastfeeding when the newborn appears to be seeking milk”, proposed in the routine mother–infant skin contact. The duration of the first lactation in the successful group was significantly longer, which was related to the start of feeding in the successful group within 1 h, suggesting that the earlier the successful breast crawl, the longer the duration of the first lactation. An EENC study indicated that the sucking duration of newborn infants in the mother–infant skin contact group was 43.58 ± 27.408 min (22), which was shorter than that in this study, indicating that breast crawl can prolong the duration of first breastfeeding. However, there is still a lack of clinical evidence that breast crawl and skin contact alone have an impact on subsequent breastfeeding of neonates, further validation is needed.

### 4.2. Effect of successful breast crawl on the ability of neonates to obtain breast milk

In this study, there were statistical differences in the BAT scale scores and degree of successful breastfeeding during the first feeding and hospitalization between the two groups. The 89.4% success rate of first-time breastfeeding was not very different from the 91.7% reported by Hou (23). Previous studies have explained the behavior and mechanism of the 9 stages of breast crawl in detail, and pointed out that this process is the sequence of fetal intrauterine motor development, and the newborn will repeatedly practice a certain behavior, so as to promote the success of breastfeeding (7). Therefore, healthy newborns can perform breast crawl after delivery as a primitive survival mechanism. Therefore, breast crawl, a two-way breast-feeding mode of autonomous breast-seeking, provides the best feeding opportunity for newborns to start breastfeeding. The 9 stages experienced by newborns in breast crawl are the integration of their neurobehavioral abilities. The repeated breast-finding process during crawl strengthens the foraging ability and optimize the ability of newborns to suck and hold nipples. Laying the foundation for continued breastfeeding. For the midwives, a very important task in this process is to protect the newborn's efforts to reach the breast, rather than human intervention to lift or rotate the newborn's body after a few attempts at crawling. Of course, in addition to paying attention to the benefits of successful breast crawl on breastfeeding, we should also consider which factors will inhibit the behavior of breast crawl within 1 h after delivery, leading to crawling failure, and take favorable measures to avoid risk factors, so as to increase the overall clinical significance of breast crawl.

### 4.3. Effect of successful breast crawl on lactation initiation

After placental delivery, the withdrawal of progesterone hormone and the increase of cortisol levels trigger the initiation of lactation, which usually occurs within 72 h after delivery and is the result of the regulation of various endocrine hormones (24), ensuring subsequent milk volume. Studies have shown that the incidence of delayed lactation initiation in China is 9.8%∼30.3% (25), which increases the risk of subsequent termination of breastfeeding. Thus, strengthening the understanding of the mechanism of inducing lactation initiation plays a positive role in preventing delayed lactation initiation. Geddes (Geddes and Perrella, 2019) and Houston (26) pointed out that lactation initiation was related to the number of prolactin receptors, in which the earlier and more frequent breast stimulation after delivery, the more prolactin receptors the newborn will have and the faster the milk production rate will be. Casavale (27) also confirmed that early breast stimulation (within 1 h) can promote the proliferation and differentiation of the mammary gland secretory cells, prevent the upregulation of the secretory cell apoptosis genes, and ensure adequate milk for follow-up. Simultaneously, continuous skin contact and neonatal sucking stimulate the peripheral nerve of the maternal nipples, promote the secretion of oxytocin and prolactin, enhance the contraction of mammary epithelial cells, and accelerate the arrival time of lactation initiation (28). In this study, 114 women (69.9%) were involved in the initiation of lactation. The number of women in the successful group was significantly higher (78.7% vs. 58.0%, *P = *0.006), and the initiation of lactation in the successful group was earlier than the failed group (*P = *0.041), which was consistent with the results of Girish (29). One of the reasons for the difference between the two groups was that the onset of breast stimulation in the successful group was earlier (median time: 51 min vs. 65 min, *P *< 0.001) and lasted much longer (median time: 60 min vs. 44 min, *P *< 0.001) than those in the failed. The second reason was that the BAT score of the successful group was higher than the failed group. Foraging, sucking, and lactating ability were exercised during crawling, which were more conducive to obtaining breast milk. Therefore, the neonatal lactation cycle is established through a successful breast crawl, resulting in strong oxytocin and prolactin responses, thereby accelerating lactation initiation and prolonging lactation (8).

Due to the influence of parity on lactation initiation (30), the confounding variable of parity was controlled, and it was concluded that successful breast crawl was more meaningful for the initiation of lactation in multipara (*P *= 0.025), but not in primiparous women (*P *= 0.143). There was no difference in the initiation time of lactation between the primary and multiparity groups. This result was contrary to the results of Huang (31), who pointed out that skin contact can accelerate the process of lactation initiation primarily because they recorded the initiation of lactation at a specific time, while this study only recorded the time range, which may have some bias. Evidence also showed that the second stage of primary labour is longer than that of multiparous labour, placental indwelling can stimulate progesterone secretion, thereby increasing the levels of estrogen and progesterone in the blood, inhibiting prolactin, and leading to slow lactation initiation (32). At the same time, the lack of lactation knowledge and breastfeeding experience, the negative emotions of fear of childbirth, and coping with lactation problems may also inhibit the initiation of lactation (33). It is suggested that the above evidence should be taken note of to increase the health guidance for pregnant women and rationally control the second stage of labour, according to maternal and foetal conditions.

### 4.4. Effects of successful breast crawl on feeding patterns within 72 h after delivery

In this study, women who underwent vaginal delivery were generally discharged 24 h after delivery without abnormalities; therefore, the rate of exclusive breastfeeding within 24 h also represents the rate of exclusive breastfeeding during hospitalisation. Meanwhile, the overall rate of exclusive breastfeeding during hospitalisation was 43.6%, which was higher than 33.3% after skin contact, as indicated in another study (34), and 37.5% according to the China Development Fund Research Society (2), but still lower than the 80% required by the review standard of infant-friendly hospitals (2018 edition). Even after 25∼48 h and 49∼72 h postpartum, the overall exclusive breastfeeding rates of the two groups (38.0% and 44.8%, respectively) did not reach this goal. Hospital settings are key to managing breastfeeding; but,under normal circumstances, women who give birth through the vagina are often only hospitalised for 24 h, and the breastfeeding instructions they receive are extremely limited.However, most feeding problems, such as milk deposition, insufficient breast milk, neonatal jaundice, and babies crying often appear only after discharge, which can make part of the mother to give up exclusive breastfeeding. Furthermore, the policy calls for exclusive breastfeeding up to six months, but obstetric responsibilities often cover only a short puerperium period (42 days) after delivery, after which it is not clear who to turn to in the event of maternal and child difficulties. The data showed that the breastfeeding rate of neonates in the successful group was higher within 48 h after delivery, which verified the results of Girish (29) and Zanardo (35), and also proved the promotion effect of early postpartum exclusive breastfeeding experience on subsequent adherence to breastfeeding (34). Feeding is the main activity of infant arousal, but lactation is not a single action, and the feeding process demonstrates a complex interaction between the mother and child. High-quality feeding interactions provide opportunities for emotional and cognitive growth while ensuring adequate nutrition, and the earlier these good interactions begin, the better the relationship (36). Breast crawl emphasises the process of how the mother holds the baby close to the breast, which is different from the action of lactation, thereby promoting the neonate to follow its instinct, in order to stimulate the breastfeeding instinct of the neonate, and to increase the number of neonates who are exclusively breastfed in the early postpartum period.

### 4.5. Effects of successful breast crawl on feeding patterns during follow-up after discharge

Feeding practices showed no difference in the follow-up data at 7 days, 42 days, and 5 months after delivery, which was consistent with the results of Gao (15) and Carfoot (37), but differed from the result of Sharma (11). The reason is that feeding methods are susceptible to interference by a variety of factors, such as access to feeding guidance, maternal mood, nutritional status, and neonate health status. The single crawling effect of the breast is not sufficient to affect the subsequent whole puerperium, and the success of the first breastfeeding does not mean that the whole feeding process can be smooth, and at the same time, different initiation and duration of mother–infant skin contact and inconsistent sample size in different studies may also lead to different results. In addition, the baseline data of the successful and failed groups may have changed after discharge.

### 4.6. Strengths and limitations

The innovation of this study lies in the fact that the existing studies on breast crawl or breast-seeking behaviour of neonates were mainly cross-sectional studies, and the only the recent clinical effects were discussed. In this study, a prospective design was adopted on a cross-sectional basis, which could provide high-quality evidence for the clinical implementation of breast crawl, especially in China. The minimal drop-out rate of our study is another strength. This study also has the following shortcomings: the sample population of this study was only from one region in China and all were full-term infants.Thus, then generalizability of the results remains to be studied; Although the effects of confounding factors have been processed according to the criteria of admittance and statistical methods, there may still be residual unknown confounding factors in the follow-up process, such as loss of follow-up bias, introduction of unknown variables, and changes in known variables.

## 5. Conclusion

A successful breast crawl means earlier initiation of first breastfeeding, which can prolong the duration of first breastfeeding, improve the ability to obtain breast milk for the first feeding and during hospitalisation of neonates, promote the successful initiation of lactation of multipara, and improve the rate of neonatal breastfeeding within 48 h after delivery. However, the long-term promotion effect of breast crawl on breastfeeding needs to be further studied.

## 6. Implications for nursing

Simply conducting a breast crawl after delivery for full-term infants and educating women about the importance of breastfeeding are not enough. The multifactorial determinants of breastfeeding require specialised medical care that are supported at multiple levels and channels, beginning in early pregnancy, and continuing throughout the lactation period. It is also necessary to empower puerperal women and their families during hospitalisation and educate them regarding the process of newborn breast crawl, so that the process can be continued after discharge, thereby strengthening the positive effects of crawling. In addition, instead of a telephone follow-up, the “Internet + “a three-way linkage continuous care model “hospital-community-family” should be adopted to empower all stakeholders and improve the overall breastfeeding rate. Besides, it can be suggested that examining the benefits of this method in late preterms can be conducted to reduce hospitalizations in late preterms due to feeding difficulties in future research.

## Data Availability

The original contributions presented in the study are included in the article/Supplementary Material, further inquiries can be directed to the corresponding author/s.
